# Systems healthcare: a holistic paradigm for tomorrow

**DOI:** 10.1186/s12918-017-0521-2

**Published:** 2017-12-19

**Authors:** Massimo S. Fiandaca, Mark Mapstone, Elenora Connors, Mireille Jacobson, Edwin S. Monuki, Shaista Malik, Fabio Macciardi, Howard J. Federoff

**Affiliations:** 1 Department of Neurology, School of Medicine, Irvine, USA; 2 Department of Neurological Surgery, School of Medicine, Irvine, USA; 3 Department of Anatomy & Neurobiology, School of Medicine, Irvine, USA; 4 Public Health Institute, Washington, DC USA; 5 Department of Economics, Paul Merage School of Business, Irvine, USA; 6 Department of Pathology & Laboratory Medicine, School of Medicine, Irvine, USA; 7 Department of Medicine, School of Medicine, Irvine, USA; 8 Department of Psychiatry & Human Behavior, School of Medicine, Irvine, USA; 9 University of California Irvine (UCI), Irvine, CA USA

## Abstract

Systems healthcare is a holistic approach to health premised on systems biology and medicine. The approach integrates data from molecules, cells, organs, the individual, families, communities, and the natural and man-made environment. Both extrinsic and intrinsic influences constantly challenge the biological networks associated with wellness. Such influences may dysregulate networks and allow pathobiology to evolve, resulting in early clinical presentation that requires astute assessment and timely intervention for successful mitigation. Herein, we describe the components of relevant biological systems and the nature of progression from at-risk to manifest disease. We illustrate the systems approach by examining two relevant clinical examples: Alzheimer’s and cardiovascular diseases. The implications of systems healthcare management are examined through the lens of economics, ethics, policy and the law. Finally, we propose the need to develop new educational paradigms to enhance the training of the health professional in an era of systems medicine.

**Electronic supplementary material:**

The online version of this article (doi:10.1186/s12918-017-0521-2) contains supplementary material, which is available to authorized users.

## Background

Human beings are complex biological systems that require coordinated, time-dependent interactions between diverse functional components for optimum survival advantage [1]. Biological systems are also directly influenced by a variety of externalities that act modify the organism’s homeostatic actuators [2]. Likewise, coordinated healthcare efforts are intimately linked to systems science, as they require monitoring of unique metrics and efficient responses to significant variations in order to operate successfully at both the individual and population levels. In fact, population health management is predicated on developing a unique understanding of how best to influence individuals, their communities, and the environment. Systems approaches, therefore, encompass an in-depth understanding of how various components interact over time.

In contrast, traditional healthcare has relied on diagnostic and treatment methods that tend to be reductionistic [3]. The patient’s presenting complaint is often analyzed in a problem-focused manner with the goal of elucidating the underlying etiology and/or pathogenic mechanism. Historically, specialty medical training and practice strive to reduce the problem to a specific organ or biochemical defect. Such an approach can fail to yield optimal results since it often ignores the important interactions between organ systems, cellular outputs and intrinsic (e.g., neural/endocrine/immune) and extrinsic (e.g., environmental chemicals, nutrition, infections, etc.) modulators. Interactions among an array of intrinsic and extrinsic modulators, however, are evident in the complex pathophysiologies underlying both Alzheimer’s disease (AD) [4] and cardiovascular diseases (CVDs) [5], wherein individual dynamic trajectories, usually unfolding over decades, underlie the transitions from at-risk, to prodromal, to manifest disease [6, 7]. Thus, focusing research efforts, drug development strategies, and healthcare approaches predicated on a single component of a system, rather than the interacting network of components comprising such a system, may obscure important etiologic principles and/or disease mechanisms, including those evident during presymptomatic stages of disease. The application of systems science [8] and its extension into healthcare, therefore, posits that health and/or disease result from the dynamical interactions of an individual’s intrinsic multiomic components (e.g., genetic, epigenetic, etc.), their resultant phenotype, and the extrinsic (environmental) factors influencing the intrinsic milieu.

**Fig. 1:**
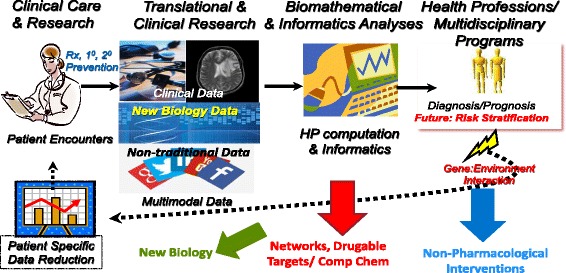
Systems Healthcare: A New Paradigm. Depicted from left upper corner and proceeding clockwise is the clinical care model with embedded clinical research that generates conventional clinical and imaging data, new biology (‘omic) data and linked non-traditional data as from social media. Data mining and reduction utilizing high performance computing and informatics analyses results in risk stratification, more precise diagnoses and prognoses, these latter reflecting gene-environmental interaction(s) and arriving at patient specific data reduction which can better inform the next cycle of encounter with the patient. In each of the colored arrows there are derivative benefits as shown in blue (non-pharmacological interventions), (new networks to discovery additional druggable targets) and green (new biological insights)

Herein, we discuss a holistic approach that encourages researchers, healthcare educators, clinicians and healthcare leaders to consider a more systems-based view of the individual (as an environmentally-influenced, complex biological system). When aggregated, such personal information may better explain population diversity (and population medicine), and thereby, help achieve more accurate diagnostic, efficacious therapeutic outcomes for all. (See Fig. 1).

## Holism and systems health

A growing evidence base suggests a potential benefit of integrative health approaches for the purposes of wellness, health promotion, and disease prevention. Integrative health approaches, as a whole, emphasize lifestyle changes, behavior modification and stress reduction which are important interventions as we identify at-risk individuals using a systems medicine approach. National survey data indicate that more people use complementary approaches to promote health and wellness than to treat a specific illness [9]. In the 2012 National Health Interview Survey (NHIS), 94% of respondents who practiced yoga and 89% of those who used natural product supplements said that they did so for reasons related to wellness; much smaller numbers used these approaches as a treatment for a particular condition [9].

Although the efficacy of integrative health in disease prevention or health promotion has not been tested widely, the case can be made for a holistic approach to health. Among the primary means to delay and prevent manifest disease are lifestyle changes that optimize individual diet, physical activity, sleep, and stress management. Sleep and nutrition are central tenants that are primarily emphasized by integrative practitioners and less so by conventional primary care providers [10]. Digestive health remains a central tenant of functional medicine [11], Ayurveda [12], naturopathic medicine [13] and traditional Chinese Medicine [14]. Finally, the holistic nature of integrative care with a mind-body emphasis often results in treatment plans incorporating psychological and somatic therapies [15, 16].

**Fig. 2:**
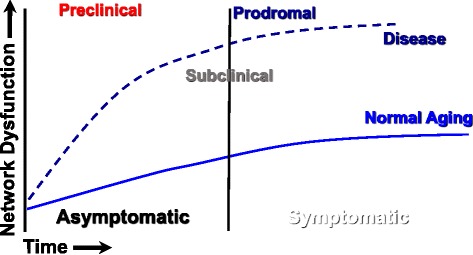
Systems view of transitions from wellness to disease. The systems view recognizes that biological networks maintain wellness but as they become perturbed through aberrant nodal and element dysfunction they drive a pathobiological process that begins with preclinical status, moving to subclinical, prodromal and then manifest disease. The distinction between periods of asymptomatic and symptomatic delimit only that individuals recognition of somatic changes but importantly the network dysfunction can substantially antedate clinical signs and/or symptoms

Holism is premised on the concept that the whole is more than the aggregation of its parts. To understand such a system, therefore, one must understand not only each individual component but also appreciate the time-dependent, inter-reliant relationships between components [17, 18]. The epochs that define the continuum of health and transition to disease are illustrated in Fig. 2, and provide a useful framework for this discussion. Formally, the system can be characterized by a description of each component, or node, and each nodal interaction, defined as an edge or element, collaborating to produce the emergent properties of the network [19, 20]. The system’s characteristics, therefore, cannot be predicted by a simple linear summation of the function of individual nodes without taking into account the complex interactions existing between them. There has been great scientific interest regarding the emergent properties of such systems, including specific output behaviors and the pursuit of insights related to the governing principles influencing the performance of such coordinated networks. When applied to healthcare, the systems approach is often referred to as systems medicine, but we expand on this concept to include the study of both structure and dynamics of interacting nodes, forming networks at multiple levels of a multidimensional matrix, including molecular, cellular, organ, person, family, community, society, and environment. For systems health, wellness, and prevention in humans, we posit that these frequent, diverse and time-limited interactions mediate change within the complex organism. Our collective challenge is to deduce the most salient nodes that underlie different states of being (healthy, at-risk, or diseased). Once these nodes and interacting edges are identified, the goal should be to beneficially modulate the network, to preserve wellness and effect secondary disease prevention wherever possible, and to mitigate pathobiology in those at greatest risk.

## Systems health in practice: Dysfunction of molecular networks

An extension of Lee Hood’s P4 medicine model [21], our P5 (Precision, Predictive, Preventive, Participatory, Personalized) paradigm involves a comprehensive understanding of the regulation and dysregulation of the complex molecular networks that forge the phenotype of an individual. In this framework, disease is a consequence of aberrant reconstitution of cellular and molecular networks that lead to organ and organismal dysfunction (e.g., the patient’s clinical presentation). The interaction of the diseased organ within the person produces a cascade of dysregulated networks, resulting in associated co-morbidities, some of which are evident and others that are asymptomatic (preclinical). In the state of wellness, networks are precisely regulated via complex homeostatic mechanisms. Through one or a series of network (or sub-network) perturbations, wellness is driven toward altered nodal activity. Such nodal modulation constitutes the at-risk state, and although preclinical, it typically provides systemic signatures, which can be discerned and quantitated, and enable detection of dysregulation during a preclinical stage. Systems level wellness, disease prevention, and health, therefore, aim to characterize specific nodal perturbations, some environmentally mediated, others rooted in the complexities of the intrinsic multidimensional networks only revealed via the aforementioned perturbations. For disease, early preclinical detection warrants attention, and likely determines the specific therapeutic intervention required to decelerate progression of the malady, or ideally, fully abrogate the network abnormalities and resultant pathobiology. Such an approach requires aggregating multi-dimensional datasets and deciphering them using high-performance computation and analytics. The goal is to determine interventions that target abnormal networks and promote systems level improvements. Such a comprehensive approach to wellness, taking advantage of multiomic data collected over time, has been attempted [22] and provides a proof of concept that requires incremental improvements. Such enhancements to our currently collected medical information will be costly and necessitate initial support from the federal government, as well as private industry, and philanthropic organizations. If and when significant progress is made in defining critical parameters of disease, which might respond to specific interdiction and thereby limit the cost of the disease itself, a convincing argument can be made to payors of healthcare services for fiscal support of such potentially life-long efforts. It remains to be determined as to how often some of these novel personal data elements should be monitored and when such monitoring should begin. What is clear, however, is that serial assessments over time, in both normal subjects and those exhibiting disease trajectories, will be necessary to begin to model both health and disease on an individual and population basis. In the following sections we consider the systems approaches to both AD and CVD, two disorders that are likely to remain significant health concerns to the world’s populations. We believe that the understanding provided by a systems approach to both of these disorders will impact our ability to prevent or mitigate their impact on human health. The potential benefits of such a systems approach include improved population health, a better patient experience, and a decrease in per capita healthcare expenditures, providing the tenets of the “Triple Aim” [23].

## Systems approaches to Alzheimer’s disease

Systems healthcare embraces the inherent multitude and often-stochastic interactions between network components, which together determine organismal functions in states of health and/or disease. Many individual disease-specific risk elements are known and used to monitor transitions to various compromised states of wellness. The finding of such individual biomarkers, such as the prostate specific antigen (PSA), or the breast cancer type-2 (BRCA2) gene mutation, enables closer scrutiny of health during the preclinical period. The more recent advances of the “omic” technologies (e.g., genomics, epigenomics, transcriptomics, proteomics, metabolomics), and their analytic methodologies, provide opportunities to extend the scope of individual elements and their relevance to health or disease. Through a combined application of biologic, mathematical, computer science, and other approaches to the interrogation of biospecimens, the breadth and depth of the complex interactions between systems (e.g., organs, biomolecules) are more readily appreciated. Such added layers of detail provide an increasingly more relevant understanding of the complexity associated with biological systems.

## Investigating Alzheimer’s disease networks

AD is the most common form of dementia in the United States [24], and around the world. AD is also the most common neurodegenerative disorder, and currently has no cure, disease-modifying therapies, or effective treatments. Age is the greatest overall risk factor, with the prevalence doubling every five years after age of 65, and eventually reaching nearly 50% prevalence at age 85 [25, 26]. Those over age 65 are projected to increase to exceed 85 million in 2050 [27]. The healthcare costs for this group alone will eclipse $1.2 Trillion by that same year. Without options to delay the onset of AD, the economic costs, healthcare burden, and social impact on afflicted individuals, their families, and society will be devastating.

If we grasp the dire consequences to the health and vitality of the world’s population posed by the lack of efficacious treatment options for AD, we must also strive to appreciate the basis for this lack of salutary success to date. We would argue that the significant setbacks in therapeutic development for AD have resulted, in part, from reductionistic approaches applied to a very complex disorder. Conditions featuring complexity are more amenable to systems biologic approaches as we will discuss in the next paragraph. We present two past approaches that are informative regarding how reductionistic viewpoints failed to adequately inform us regarding AD therapeutics.

First, until recently, patient selection for therapeutic AD trials was predicated on the presence of obvious clinical manifestations of disease, as either mild cognitive impairment (MCI) [28] or AD [25]. Waiting to intervene until the symptomatic stages of AD, however, may not be optimal for therapeutic efficacy. Associated with the presence of clinical manifestations of AD, the neural substrate may be sufficiently damaged as to be incapable of an efficacious response to a therapeutic. More recent recommendations for clinical trials [29] have encouraged the introduction of drug therapies during the preclinical (i.e., asymptomatic) stages of AD, to take advantage of a less impaired and potentially more receptive neural substrate. The latter approach requires the development and availability of biomarkers that accurately classify individuals at-risk of AD, well in advance of clinical manifestations. In addition, despite a wealth of preclinical and clinical information showing marked etiologic differences, a clear distinction has not been made in the development and testing of therapeutic options directed towards the two major forms of AD, the early-onset AD (EOAD) and late-onset AD (LOAD). While both disorders feature similar end-stage elements in brain neuropathology (e.g., plaques and tangles) [30, 31], they differ significantly in their primary etiologies and clinical trajectories [32, 33]. Specifically, most cases of EOAD are relatively rare (~5%), present symptomatically prior to age 65, typically include mutations within one of three genes (PSEN1, or the presenilin-1 gene; APP, or the amyloid precursor protein gene; or PSEN2, or the presenilin-2 gene), and have a familial predisposition, usually expressed in an autosomal dominant manner. In contrast, LOAD is the much more common (~95%) sporadic form that usually presents after 65 years of age, has no sole genetic basis or familial predilection, and features a pathobiologic profile suggestive of genetic predisposition influenced by environmental (epigenetic) factors. While certain transgenic animal models provide adequate surrogates for genetic forms of EOAD [34, 35], relevant models for LOAD do not exist.

A reductionist approach would have concluded that since amyloid beta and tau protein accumulations are end-stage hallmarks in brains of both EOAD and LOAD, regulating the accumulation of these molecules might lead to effective therapeutics for AD. Such agents were formulated, tested in transgenic animal models, and noted to attenuate the amyloidogenic processes, similar to what was featured in genetic forms of EOAD [36]. The vast majority of individuals treated in clinical trials, however, suffered with symptomatic stages (MCI or AD) of LOAD. As a result of such reductionistic thinking, therapeutic interventions provided efficacy measures that were either unimpressive or completely lacking during late-stage clinical trials [37].

A holistic approach to AD will likely result in the development of preclinical treatment options that would be based on specific differences in pathobiology for EOAD and LOAD, lying upstream of the end stage neuropathology featuring accumulations of amyloid and tau. Systems biological approaches to AD require the willful integration of diverse sets information to formulate a better understanding of the complex disease state(s). The enormous information gained through use of “omic” technologies is at least in part due to the integration of orthogonal data, providing an improved appreciation for network functions, including their complex and often unobservable interactions. The generated “omic” information’s representation of a complex network has a basis in mathematical/computational sciences, including graph theory [38]. Specific to biologic systems, the acquired disparate pieces of information are ultimately formulated to provide a clearer understanding of a complex health state (e.g., EOAD or LOAD). The distinction between reductionism and holism, therefore, is not nuanced, with the latter potentiating the definition of drug targets, the disease-specific role or function of a gene or metabolite, and/or diagnostics for otherwise asymptomatic disease states.

The following “omic” methods provide examples of how systems (network) biological principles have been and continue to be applied to the study of AD. While individual “omic” methods provide a substantial view of altered networks within a system, the ultimate power of these technologies will be recognized once a more facile integration of “multi-omic” data becomes mainstream.

## Current approaches to Alzheimer’s disease - genomics

The use of high throughput DNA sequencing to screen patient-derived DNA for disease-associated alleles and deriving a genetic risk assessment is evolving beyond the laboratory. Currently, the lay public is increasingly able to access this technology through a variety of for-profit companies that deliver potentially actionable information regarding individual risk for certain diseases. Genome-wide association studies (GWAS) have used large numbers of subject samples (cases and controls) to identify single nucleotide polymorphisms (SNPs) that may be specifically related to disease. 23andMe (http://23andme.com), for example, offers low cost, direct-to-consumer genomic testing and interpretation. As of 2017, 23andMe provides analytic options on saliva specimens that inform the individual regarding not only genealogy, but also a limited number of health risk analyses for up to 10 diseases and health conditions. Duplication of the APP genetic locus, for example, has been confirmed as a cause of autosomal dominant EOAD [39]. The specificity of the APP locus in disease etiology has been additionally supported and further detailed by the discovery of a protective mutation within that gene (A673T), which reduces amyloid beta production, as noted in a small Scandinavian population that does not suffer with AD [40]. Together with age, the best-known risk factor for LOAD is inheritance of the apolipoprotein E (APOE) E4 allele, with a single genetic copy increasing the odds of developing LOAD in a normal lifetime by 2–4 times, while two allelic copies provide more than 8 times the likelihood [41]. While evaluations of large monogenic pedigrees helped confirm three highly penetrant autosomal dominant genes as responsible for the vast majority of EOAD cases [42], GWAS has provided evidence for over 20 susceptibility genes in LOAD [43–46], most of which show significant statistical associations back to the neuropathology [47]. Some of the genetic contribution remains unaccounted for [48], although pleomorphism of individual loci [49], gene-environment interactions [50], and epistatic gene-gene interactions [51] could account for much of this “missing heritability.” Indeed, recent assessments of Alzheimer’s Disease Genetics Consortium (ADGC) datasets identified numerous SNP-SNP and gene-gene interactions among LOAD genetic loci [52], and systems analyses [53] in LOAD subjects have defined specific genetic nodes, edge interactions, and network perturbations that may eventually elucidate the associated complex pathobiological mechanisms. Such in-depth understanding of the network biology and functional gene modules [53–55] will provide a better opportunity for therapeutic breakthroughs in related to LOAD (and also CVD) [56].

With the capabilities provided by the Alzheimer’s Disease Neuroimaging Initiative (ADNI) and other collaborations, AD genetics coupled with neuroimaging have provided tremendous momentum to the field, with ADNI alone being listed as a corporate coauthor on >100 PubMed citations per year since 2010. Relevant examples include APOE4 linkage to fMRI and other imaging findings years before AD onset [57] and linkage of new GWAS-confirmed loci associated with reduced hippocampal volume [58]. ADNI cohorts have also been used for multimodal analyses to define many new putative imaging biomarkers of AD and MCI, with numerous SNP associations, outnumbering those linked to neuropathologically-defined AD [59]. In a recent example [60], a continuous polygenic hazard score for age-specific AD risk was derived using GWAS SNP (IGAP) data, APOE status, and population-based AD incidence rates in an ADGC cohort, and then tested in independent postmortem (NACC with neuropathology) and premortem cohorts (ADNI with CSF biomarkers). This polygenic hazard score correlated well with neuropathologic (Braak stage and CERAD score), cognitive (CDR-SB), CSF (AB42 and total tau), and imaging biomarkers (entorhinal cortex and hippocampal volumes) [60]. Unfortunately these genomic approaches remain reductionistic and require addition multiomic input variables, along with additional genomic data inputs, to provide more holistic assessments of AD risk in clinical trials and routine patient care.

## Current approaches to Alzheimer’s disease – Metabolomics

Metabolomics investigates alterations in the quantities of the small molecules derived from anabolic or catabolic processes, and are detectable within biofluids (blood, urine, saliva, cerebrospinal fluid), cells, tissues, and organs. The most commonly used technologies for metabolomic analyses currently include (a) mass spectrometry, featuring electrophoretic or chromatographic (gas- or liquid-based) separation methods, and (b) NMR (nuclear magnetic resonance) spectroscopy methods. Metabolomic analytic technologies have advanced significantly over the last decade and provide an ever-increasing assessment of complex biological systems. Metabolomic analyses detail the downstream consequences of disease, providing network information proximal to the clinical phenotype. Such information is in contrast to genomic or other “omic” analyses that provide information that is generally considered upstream from the phenotypic manifestation(s). Despite the tens of thousands of substances available for quantitative and qualitative analyses using metabolomic methods, many additional compounds have yet to be fully integrated into pathways and/or definitively identified (or annotated) [61]. Direct analysis of brain tissue provided the earliest evidence of metabolomic dysregulations associated with AD [62], with notable reductions in certain sphingolipid species. Since then, our group [63–65] and others [66–70] have confirmed the dysregulation of lipids and other metabolites, within brain and in the periphery, which may be relevant to the pathobiology of LOAD, and even herald the phenoconversion from health to disease. Despite these advances, analyses using metabolomics alone do not approach the holistic assessments required for comprehensive risk assessments. Metabolic network abnormalities are associated with AD (and other disease states), but multimodal systems approaches are needed to understanding the unique interactions between metabolic pathways, other “omics”, and external environmental influences. Such methodologies will ultimately contribute to the improved definition of clinical phenotypes via combinatorial network approaches that provide a more holistic view of health and the transition to AD.

## Holistic approaches to Alzheimer’s disease

Understanding the basis of health and disease through systems biological methods may identify a range of individual life-style choices that can mitigate AD risk. A prime example comes from the link between metabolism and the genome, through epigenetic modifications [71, 72], and especially DNA methylation [73, 74]. While metabolism’s effects on epigenetic regulation remain a complex field of inquiry for most, the positive health effects of a Mediterranean diet [75] and exercise [76, 77] are well recognized, despite limited adoption.

Lacking therapeutics, behavioral modifications may ultimately provide the best individual disease prevention options, especially if adopted as a life-long health strategy. In other words, a key component to controlling personal health in relation to AD is linked to human diet and exercise [78, 79]. Specifically, moderate exercise from mid- to late-life is associated with lower dementia risk [80]. A high fat diet on the other hand is known to disrupt circadian clocks [81, 82], alter the gut microbiome [83], and thereby negatively impact metabolism [84] in association with AD. In contrast, an increase in specific dietary fatty acids and other nutritional supplements may prove beneficial in slowing the progression of AD in animal models [85], and when administered to humans during early clinical stages of LOAD [79, 86]. The latter nutritional support is thought to provide substrates for synaptic resuscitation, with documented improvements in memory performance [87]. Reductions in the availability of similar substrates in human plasma have been associated with preclinical and clinical stages of AD [63, 69, 70].

**Fig. 3:**
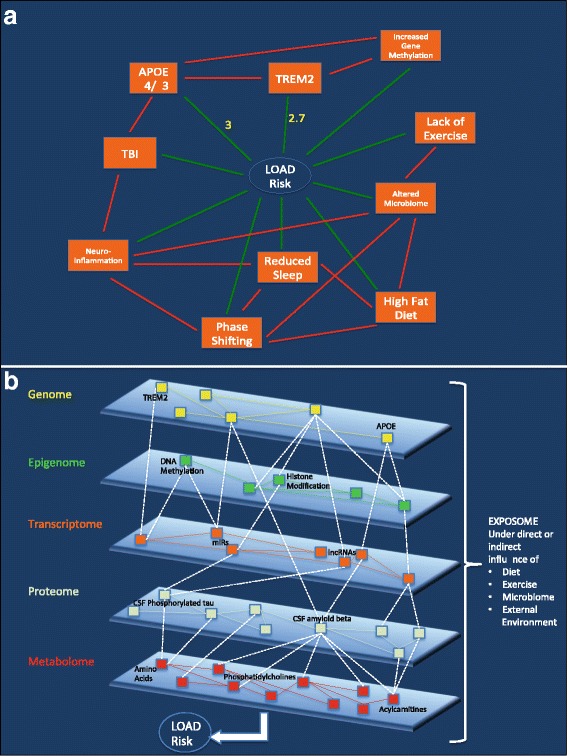
Systems biological framework associated with example case. Panels **a** and **b** represent the 2-dimensional (2D) and 3-dimensional (3D) depictions, respectively, of the relevant internal and external network architecture associated with the case. (**a**) The clinical features associated with our subject are represented in 2D as nodes (orange rectangles) and edges (lines) directly connecting the nodes to LOAD risk (green), and interacting with other nodes (red). Yellow numerals express known risk relationships between specific nodes and LOAD risk. Note that the majority of edges have no numerical representations and that not all nodes are shown to interact via edges. Edges represent either uni- or bi-directional relationships between nodes, and can provide positive or negative influences. (**b**) Our example case is now represented in 3D, displaying proposed more holistic relationships for our example subject’s personal multiomic profile and LOAD risk. Note the complex interactions between nodes (small colored rectangles) in each layer of the individual’s intrinsic multiomic matrix, including features specific to this case (in bold black letters – APOE, TREM2, DNA methylation) and other features not specific to this case (in black letters). There are complex, yet to be determined relationships represented within each “omic” layer (solid color edges between nodes), as well as relationships between nodes of alternative “omic” layers (dotted white edges). Together, these nodes and edges embody a multidimensional representation of a complex system of interactions that 1) are unique to the individual, and 2) are under constant influence by external (environmental) factors (i.e. the exposome) acting on various layers of the internal multiomic matrix, in reducing or increasing LOAD risk. LOAD = late-onset Alzheimer’s disease. APOE = apolipoprotein E gene. TREM2 = trigger receptor expressed on myeloid cells 2 gene. CSF = cerebrospinal fluid. miRs = microribonucleic acids. lncRNAs = long non-coding ribonucleic acids

Ultimately, direct comparisons between yet to be developed efficacious therapeutic agents and the aforementioned lifestyle modification will be tested. A systems biology perspective, taking into account the variety of intrinsic and extrinsic factors associated with health and disease, will likely provide the identity of future therapeutic targets required to diminish or prevent AD. Current systems biology focuses on connectivity mapping to find theoretical and/or functional relations between network nodes made up of genes, proteins, and small molecules, all sharing a mechanism of action, a physiological process, a disease, or specific drug target(s) [88]. Starting with a research objective, such as finding novel network interactions within a transcriptomic, proteomic, or metabolic pathway, investigators can begin to construct complex networks of genes, proteins, and metabolites to investigate novel interactions within such “multi-omic” networks. Such relational information is being added to evolving databases that include both empirically validated interactions and those resulting from computational predictions. From these approaches, therefore, empirically active and theoretical networks can be described, and novel disease-related targets discovered and tested. Connectivity maps, such as developed by the Broad Institute and Harvard [89], link gene patterns associated with disease to corresponding patterns produced by drug candidates, thereby allowing researchers the opportunity to screen compounds against genome-wide disease signatures rather than a pre-selected set of target genes [90–93]. A multiomic approach (Fig. 3) to disease and personalized risk assessments remains in the nascent stages of development, despite the growing interest defined by the number of recent publications. Specific considerations are provided as a detailed Case Example and analysis (see Additional file 1), which considers personal intrinsic and health information and extrinsic influences that may eventually be used to develop a LOAD risk profile. Similar strategies may ultimately be applicable to other conditions but may ultimately provide health guidance approaches that empower individuals to maintain states of health and minimize factors associated with disease.

## Systems approaches to cardiovascular disease

The value of a systems healthcare approach can be further evaluated in the context of CVD. Despite advances in diagnosis and treatment, CVD, and specifically coronary artery disease (CAD), remain the leading cause of mortality and morbidity in both men and women in the world. CAD is a heterogeneous disease entity with a broad range of outcomes that develops over a long period of time, commencing slowly through a prodromal stage of about 30–50 years, followed by a fast expanding asymptomatic period of about a decade and then rapidly progressing to a clinical stage with symptoms [94, 95]. Macro-environmental factors such as lifestyle, stress, pollution, as well as social determinants of disease interact with genomic variations to predispose an individual to early stages of disease. Micro-environmental factors such as inflammation, lead to expression of cellular signals that then regulate disease progression [96].

Although CAD events have declined in the past decade, recent data suggest that this trend may have reached a plateau and in fact, most recently, an upward swing in CAD deaths has been observed [97]. The reductionist approach, described above in earlier sections, has led to breakthroughs in clinical treatment of CAD. However, even in individuals who are optimally treated for traditional risk factors, residual risk of incident CAD events and disease progression persists [98, 99]. In order to reduce residual risk and improve CAD outcomes, as in other chronic diseases, a holistic systems medicine approach which examines relationships among identified risk factors as well as effect of novel pathways using an interconnected framework of genetic, molecular and environmental factors is needed [100, 101]. The holistic approach of systems medicine has the potential to describe more precisely the complex clinical CAD phenotype in a given individual, leading to not only earlier subclinical disease detection, but also more effective and directed therapy, thereby eliminating residual risk and improving outcomes.

## Current approaches to cardiovascular disease – Genomics and epigenetics

Representing tremendous heterogeneity, CVD includes both monogenic and polygenic conditions. The CAD loci identified by GWAS are mainly associated with the early stages rather than the later phases of the atherosclerotic clinical disease phenotypes. Meta-analyses of GWAS through the CARDIoGRAMplusC4D consortium have now identified 152 susceptibility loci for CAD [102–104], shedding light on a number of novel biologic pathways involving genes that appear to be operating in the vessel wall or in the early atherosclerotic course rather than later phases of the atherosclerotic clinical disease phenotypes [105, 106].

Due to the small effect size of each individual SNP, the clinical utility of individual SNPs to predict disease likelihood is quite modest [107]. As a result, the concept of Genetic Risk Score (GRS) has been utilized; using either weighted or unweighted SNPs to generate a single aggregate score to assess predictive value for long-term CVD events [108]. The GRS has incremental predictive value and clinical utility for incident CVD events, beyond traditional risk factors, showing a heritable component attributable to the multiple independent or interacting variants [109–116]. In the Myocardial Infarction Genes (MI-GENES) clinical trial a CAD GRS was incorporated into a conventional risk prediction algorithm. Informing study participants of their genetic risk for CAD was associated with lower LDL cholesterol levels than disclosure of clinical risk factors alone [117]. Knowledge of an underlying genetic predisposition to common polygenic CAD may prompt both physicians and patients to more aggressively address modifiable risk factors before disease onset.

The GWAS approach, using genetic variance alone, cannot recognize and explain the pathological changes and clinical progression of CAD phenotypes [118]. The atherosclerotic process is a complex phenomenon involving epigenetic adjustments that are adapted and programmed to various gene expressions. Mechanisms related to epigenetics or modulations of gene expression include methylation and histone modifications many of which are triggered by lifestyle factors. Epidemiological and clinical trials have established that various risk factors like cholesterol, hypertension, diabetes, smoking and unhealthy lifestyle behavior are associated with atherosclerotic plaque pathogenesis and gradual progression to atherosclerotic disease. In a recent study examining genetic and lifestyle factors in 55,685 participants, in those with high genetic risk, as assessed by a 50 SNP GRS, a healthy lifestyle resulted in a nearly 50% lower risk of CAD, showing that epigenetic changes triggered by lifestyle can result in resiliency in the face of adverse genetic risk [119].

## Current approaches to cardiovascular disease – Metabolomics

Metabolomics studies have begun to reveal previously unknown factors that may contribute to the mechanisms and pathogenesis of CAD (and other human disorders), including dietary and gut microbiome variation and potential links between them. As an example, a high fat diet appears to alter synchronization of the circadian clock [81, 82], and also impact the gut microbiome [83]. In turn, the gut microbiome directly influences oscillatory transcriptional programs in the liver [84], evidenced through metabolomic analyses. Profiling of blood metabolites could have an important role in predicting or monitoring subclinical atherosclerosis and identifying patients at risk for early CAD. A number of circulating metabolites, like the trimethylamine-N-oxide (TMAO) and lysophosphatidylcholines, are considered potential biosignatures for increased risk of cardiovascular incidents [120, 121]. Specifically, TMAO is produced by the interaction of the gut microbiome with phosphatidylcholines and carnitine in the diet, and are present in higher concentrations in a meat-based cuisine [122–124]. In addition, a multicohort epidemiological study examined 68 plasma metabolites and indicated that higher phenylalanine and monounsaturated fatty acid levels were associated with higher CVD risk, and conversely, higher omega-6 fatty acids and docosahexaenoic acid (DHA) levels were associated with lower risk of CVD [125].

## Holistic approaches to cardiovascular disease – Imaging, information integration, and beyond

To fully implement a systems medicine approach, accurate assessment and phenotyping of subclinical disease is critical (see Fig. 2). Cardiac computed tomography (CT) offers coronary calcium scoring or CT angiography to assess calcification of the arteries, which is correlated with plaque burden and an accurate measure of subclinical disease. Optical coherence tomography (OCT) is being used to measure the lipid and macrophage content of arterial plaques and give insight about plaque composition and stability. The development of chemical or biological probes, and imaging agents in animal models, that sense molecular pathway alterations, allow monitoring of such dysregulations in CVD. Magnetic resonance imaging, fluorescence imaging, bioluminescence imaging, positron emission/single photon emission computed tomography (PET/SPECT), and ultrasound are techniques that take advantage of molecular probes designed to image enzymes, receptors, endothelial cells as well as the biological processes of apoptosis, angiogenesis and thrombosis [126]. Positron emission tomography (PET) using 18F–fluorodeoxyglucose (FDG), which is stored in metabolically active cells, can mark inflammatory networks involved in the myocardium and the vasculature. In the systems medicine approach, the use of advanced imaging techniques as an adjunct to omics technologies allows not only improved definition of the CAD phenotype, from those that have only prodromal disease to those with the highest vulnerability, but also permits monitoring of response to therapy and disease progression.

**Fig. 4:**
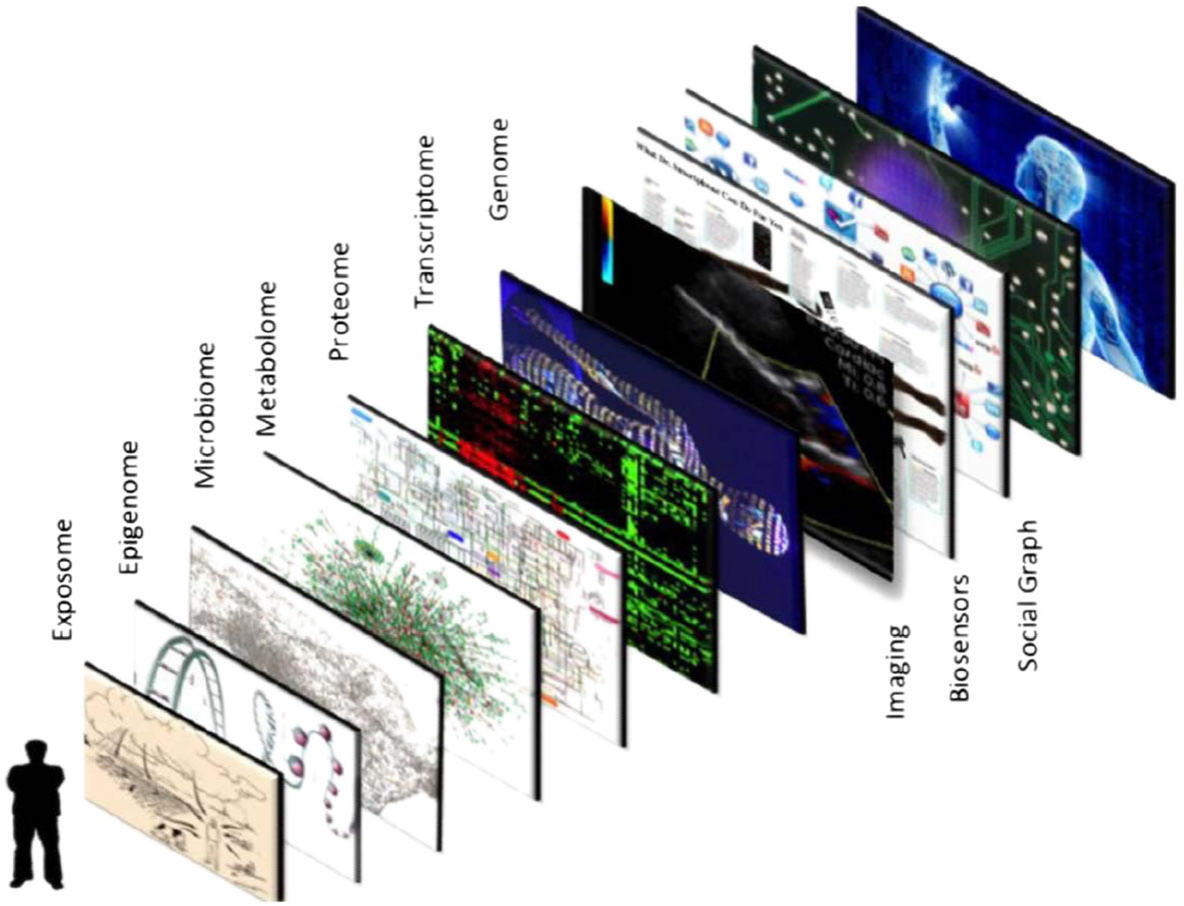
Connecting the exposome to the readout of the genome. Given that most major human diseases are genetically complex is it necessary to characterize the extrinsic (environmental) exposures (exposome) and ingestion nutrients (acting through the microbiome) each capable of producing epigenomic modifications that alter the transcriptions in multiple visceral organs and CNS or the gastrointestinal tract (microbiome, innate immune signaling and afferent activation of enteric nervous system). *“Figure reprinted from Cell, Volume 157, Issue 1, / Eric J. Topol, Individualized Medicine from Womb to Tomb, Pages 241-253, 2014, with permission from Elsevier”* [127]

The layering of omics and imaging technologies described above form part of the data that is needed for a true grasp of the biosignature of an individual. In addition to these technologies, Topol describes layering of other technologies to capture a “panoramic” view of individual’s health, including layers of data from biosensors, social media, as well as the exposome or environmental exposure data (Fig. 4) [127]. This level of integration will require not only storing or retrieving information from a central repository, but also automated analyses.

The Trans-Omics for Precision Medicine (TOPMed) research initiative developed by the National, Heart, Lung, and Blood Institute will “couple whole-genome sequencing (WGS) and other -omics data with molecular, behavioral, imaging, environmental, and clinical data from studies focused on heart, lung, blood and sleep disorders” [128]. Phenotypic, genomic, behavioral and socioeconomic data from existing cohort studies (e.g., Framingham Heart Study and the Jackson Heart Study), will be combined in a novel manner. The use of these integrated data platforms may allow a better understanding of the systemic interactions among comorbidities, lifestyle, and socioeconomic backgrounds that impact CVD outcomes. A systems medicine framework is starting to be utilized in clinical investigations as well. The American Heart Association’s recently funded FAMILIA study targets low-income, underserved, at-risk families in Harlem, NY to understand the impact of family-based lifestyle intervention on behavioral risk factors among parents/caregivers and preschool-aged children [129]. In addition to using genomic and molecular data, this study will assess behavior and lifestyle across the family unit. The goal of the study is to identify the genetic, genomic, and molecular signature of favorable versus poor responders to lifestyle intervention, thus permitting future tailored approaches, as well as identify novel therapeutic and diagnostic targets in network models of early atherothrombotic disease.

As data from these basic science, epidemiologic, and clinical research efforts become available, a systems medicine approach, becomes more plausible on a population-wide scale. Once adopted, systems medicine will result in a significant paradigm shift from acute intervention to prospective, holistic and personalized cardiovascular health care. It will allow rapid identification of individuals predisposed to illness, classifying disease on a molecular basis to improve diagnostic and prognostic precision, discovering predictive pharmacogenomics profiles, and developing non-invasive imaging methods to detect disease and monitor response to therapy with the ultimate goal of improving quality of life and longevity.

We anticipate that specific systems approaches to both CVD and AD, including modulatory external influences on the individual’s intrinsic “omic” functions, such as through improved lifestyle choices, may provide a positive impact over the short term in the majority of individuals at risk of disease. Ultimately, the development of relevant biomarkers, point of care technologies, and wearable sensors to assess, process, and provide useful feedback to the individual, in real-time, will eventually lead to modifications of behavior and risk that will benefit patients. It is through the perpetual integration of these data elements that the strength of systems biological approaches will be realized.

## Systems healthcare – Policy, ethical, and social issues

Systems medicine provides a promise for significant gains in the diagnosis and treatment of disease and the delivery of healthcare. At the same time, however, it also poses substantial challenges to full implementation, including current policy and regulatory dynamics, concerns regarding data and privacy, issues surrounding access and cost, and the difficulty of accounting for the external factors outside of direct medical care that influence health.

## Systems healthcare – Policy landscape and specific issues

Continued uncertainty in the U.S. federal policy landscape that surrounds healthcare holds broad implications in the transition to a true systems medicine approach. Since the Affordable Care Act (ACA) was passed into law in 2010 [130], the House of Representatives voted over 60 times to amend or repeal the law, including repealing provisions that attempted to shift towards more preventive care and payments that focus on health outcomes and care coordination. While recent versions of repeal failed in the Senate [131], there is a chance for continued uptake of this type of legislation, and executive and administrative action continues to dismantle the current law.

Worth highlighting for this discussion is the significant reduction in prevention programs and coverage requirements that would take place if legislation similar to that passed recently by the House of Representatives were to be signed into law at some point in the future. Such a law would eliminate the Prevention and Public Health Fund, which is now 12% of the Centers for Disease Control and Prevention’s (CDC) base budget. In addition, states would have the ability to waive out of the requirement that insurance plans in the Marketplace must cover a package of ten Essential Health Benefits [132], including prevention and wellness services and chronic disease management. The lack of awareness of the importance of prevention programs and their impact on overall health suggests a difficulty in translating a true predictive and personalized medicine approach on the federal policy level. Indeed, while “innovation” seems to gain traction, translating that to broad-reaching prevention implementation has proven to be a harder sell.

Despite the President’s Fiscal Year 2018 Budget that requested deep cuts to federal non-defense discretionary spending that funds both science and innovation, including at the National Institutes of Health (NIH) and the CDC [133], innovation and “cures” continue to be a priority of the legislative and administrative branches, where now “predictive analytics” are discussed as a way to reduce costs while improving care delivery and management and cracking down on fraud. The twenty-first Century Cures Act [134], for example, signed into law in 2016, authorized $6.3 billion in funding (including $4.8 billion to NIH) for precision medicine and biomedical research. Some of those funds targeted Vice President Joe Biden’s Cancer Moonshot program and President Obama’s Precision Medicine Initiative (PMI). While this money is only authorized (not appropriated yet), funding commitments via the appropriations process has already moved forward. In order to offset, or “pay for” the bill, however, Congress tapped into the Prevention and Public Health Fund, arguably the only source of funding that was originally targeted towards innovation in the public health sector. Scientific innovation without a pathway for implementation makes it difficult to realize the extent of such innovation.

The PMI is an enterprise of the National Institutes of Health (NIH), spearheaded by the University of Pittsburgh, and launched by President Obama in 2015. There are two main components associated with the PMI. The shorter-term focus is on cancer while in the longer-term goal involves viewing health and disease in a broader framework of understanding the risks and mechanisms of disease and predictive therapies reach broad-scale impact [135]. A small-scale rollout, recruiting 10,000–15,000 of the eventual 1 million participants and a national rollout is planned for next year.

Furthermore, Healthy People 2020 included a new Genomics topic area with a goal to “improve health and prevent harm through valid and useful genomic tools in clinical and public health practices” [136]. The objectives are based on recommendations from the U.S. Preventive Services Task Force (USPSTF) and the Evaluation of Genomic Applications in Practice and Prevention Working Group (EGAPP), each focused on discrete diseases with evidence that early screening or intervention could improve broad-scale health outcomes. While genomics is just one piece of a fully integrated systems medicine approach to healthcare, the recognition that genomic-level data could improve population level health is significant and a necessary step towards a systems medicine implementation.

It is now widely recognized that there are numerous causes of disease and pathways that impact our ability to be and stay healthy. These select examples are not exhaustive, rather a sample to illustrate how there is consideration of the underlying components of disease and efforts towards understanding how to prevent and treat disease even at the federal policy level which tends to be slower moving and less nimble than the private sector.

## Systems healthcare – Data, privacy, and discrimination

A systems medicine approach requires storing and sharing a significant amount of very sensitive information with many types of health professionals and a variety of administrative systems, all of which raise a host of privacy concerns. In most circumstances, formal data sharing plans will be required, including how best to protect protected health information (PHI). The vast amount of data required to harness systems medicine raises important questions, such as: who owns the data, where is it stored, and who has access to it? The current realm of “Big Data”, especially the immense amount of digital information now stored and readily accessible electronically, is directly linked to evolving systems medicine approaches. Big Data is now using sophisticated analytics to predict what an individual’s future healthcare needs might be, based on interaction with providers, medical history, internal “omic” measures, and external social and environmental influences. The collected information might also include an individual’s presence on social media [137]. The vast amounts of electronic health records now available contribute to massive datasets featuring clinical information, demographics, and treatment course of individuals that are prime for data analytics [138], but must be safeguarded from intrusions [139]. Recent hacking of large digital databases [140] should provide the impetus for improved security going forward. A significant number of systems, however, are not online or are not easily integrated with other systems, providing data fragmentation and limited utility. Without the opportunity for all clinical records and data on varying systems to be linked to one-another, systems medicine will be hard to implement.

More public and private payers are harnessing Big Data in healthcare but the privacy concerns surrounding these advanced algorithms remains worrisome, as described above. Current privacy structures – such as the Health Insurance Portability and Accountability Act (HIPAA) [141] and anti-discrimination laws such as the Genetic Information Non-Discrimination Act (GINA) [142] are limited and likely not suited for an environment where the scope of PHI has broadened so significantly. Security breaches of PHI from insurance companies are devastating; expanding the scope of information collected will likely regulatory structure to ensure privacy and protection of sensitive data. Additionally, the fluid nature of real-time data collected and stored means that security risks must constantly be updated.

The use of data for personal decision-making is a significant factor as the pace of our technological advances increases. If we can identify the disease-specific biomarkers, and predict the disease onset, how much does an individual want to know about their disease risk and when? Would the answer change if the identified disease has no known cure or any treatment to slow its progression? Conversations on this topic need to be expanded and include all of society. In addition, new technologies are being developed that can create genetic changes or “edits” that can be passed on to future generations [143]. To what end could we choose to edit what is inherited and what is not? How can we predict the unintended consequences of tinkering with the genetic code, such as accidental gene mutations? [144, 145].

More recently, under the guise of restoring internet freedom, the U.S. Administration has proposed changes to net neutrality rules that have significant implications on health care. The proposed rules may result in broadband providers charging more for different levels of connectivity, which could leave smaller healthcare practices and rural hospitals unable to afford a fast connection, hampering their data collection ability [146]. Additionally, the development and usage of health apps could be impacted, which would make disease monitoring and behavior change programs less effective.

## Systems healthcare – Access and cost

New and innovative technologies will always bring about issues of access and equity. How do we ensure that the latest and greatest is available to everyone? If a new treatment is expensive, insurance companies may only cover a portion of it, which means that only those individuals that can afford to pay out of pocket realize the benefits; increasing existing disparities in health outcomes. Payers haven’t yet begun to fully embrace genetic testing, one of the multiple components of a systems medicine approach. To truly make progress in population health through a systems medicine approach, we need to understand how to prevent and reduce the onset of chronic diseases, particularly in underserved populations. How can we guarantee equal access to new diagnostic techniques and therapies when millions of Americans aren’t even receiving basic primary care? While the cost of sequencing the human genome has decreased significantly over the last decade [147, 148], the cost to maintain the massive datasets of information that require constant updating can be cost prohibitive [149].

## Systems healthcare – Social determinants

Often disregarded in genomics-level discussions on disease and health are the external variables, or social determinants, that influence our health that a systems medicine approach would incorporate. These include our environment – the ability to breath air free from pollutions and maintain an active lifestyle with walkable green spaces; access to healthy foods; education; employment status; socioeconomic status, among others, as well as how each of these variables influence one another across the life-span and generations. Definitions of the social determinants of health vary from the broad: “any nonmedical factors influencing health” and “not controllable by the individual but affect the individual’s environment”; to more specific “the conditions in which people are born, grow, live, work and age, and which are shaped by the distribution of money, power and resources at global, national and local levels” [150]. While there is an increasing recognition that nonclinical factors have a major impact on people’s health, measuring outcomes – particularly those that might not realize results for years to come or involve significant investments to maintain – can be a challenge.

Progress is being made to harness all factors involved in the health of an individual. Evidence based interventions can address common risk factors for multiple disease states, but a mere 3 % of federal health spending is currently directed towards preventing an illness before it occurs. Even less is attributed towards these non-medical causes of poor health and disease. To truly “bend the cost curve” and ensure robust health solutions for all, external factors – where we live, work, and play need to be addressed.

## Systems healthcare – Economic implications

The returns to a systems approach to healthcare should extend beyond improved quality of life and increased longevity for individuals. In particular, a systems approach – through its promotion of population-based wellness and prevention – has the potential to generate meaningful savings of both public and private health care expenditures and have beneficial spillovers to the family members and friends who help care for the sick. A systems approach to Alzheimer’s disease and secondary prevention of cardiovascular disease offer illustrative cases in point.

In 2010, annual health care costs attributable to dementia, i.e., net of the financial burden of other co-occurring diseases, were between $41,000 to $56,000 per person or $159 billion to $215 billion in aggregate [151]. About three-quarters of these costs are from institutional and home-based long-term care [151]. Although these estimates place a monetary value on informal care provided by family members and friends, they do not account for the substantial non-monetary costs to caregivers in terms of negative consequences to social, physical, and psychological well-being [152, 153]. To the extent that a systems approach to Alzheimer’s disease can delay disease onset by even a couple of years, it has the potential to save hundreds of billions in direct health care costs and even more in terms of improved well-being for caregivers.

Treatment for cardiovascular disease (CVD) accounts for about 1 out of every six health care dollars or about $313 billion annually [154, 155]. Numerous reviews have shown that secondary prevention of CVD, particularly disease management programs that promote lifestyle change (e.g., smoking cessation and exercise), medication adherence and multidisciplinary team-based care, can improve quality of life, reduce hospital admissions and lower health care spending [156, 157]. Transitional care models that foster secondary prevention have in fact been shown to be cost saving [158, 159]. To the extent that secondary prevention is integral to a systems approach to CVD, it has the potential to save billions of dollars annually.

## Conclusions

To integrate systems approaches into clinical practice, emerging and current healthcare workers must be exposed to new and different training programs. Training would involve scientists, clinicians, and other providers who embrace holistic approaches that encompass care for the whole person (mind, body, and spirit) and incorporate these with information technologies. Fully-integrated systems training would therefore involve a broad range of healthcare professionals, including nurse practitioners, clinical psychologists, nutritionists, and licensed providers of alternative therapies (naturopathic doctors, acupuncturists, yoga instructors and massage therapists), providing each with foundational knowledge about each others’ disciplines and to enable ideal education on behalf of patients [160]. This integrated, trans-disciplinary approach to training is also seen in the biopsychosocial model of disease, wherein dimensions of behavior and social milieu are recognized along with biology [161]. Ultimately, such training would lead to collaborative team-based coordinated care that will best serve our patients.

Relatively few “best practices” for such training have been established, but some are emerging. The Coordinating Action Systems Medicine (CASyM) [162] established a European plan for systems medicine education that encompasses the following goals: (1) establishing frameworks that span all aspects of medical education and all relevant disciplines; (2) facilitating courses on “traditional” topics that incorporate dynamic systems approaches and visualization-based gadgets; (3) educating research physicians and clinical practitioners more thoroughly in statistics, bioinformatics, omics technologies, and modeling for medical purposes; and (4) adapting software for practical usage by clinicians. A number of US academic institutions have initiated programs relevant to systems medicine given the training imperative [3, 163, 164], with longitudinal training being an important consideration given the longer cadence of learning compared to other clinical areas [135]. Different department, school, and center-based programs have been initiated for pre-doctoral and post-doctoral trainees [3, 164], with NIH CTSAs, systems biology centers, and ACGME fellowships in clinical informatics (instituted in 2014) serving as additional seeds or focal points for interdisciplinary training. K-12 [165] and CME programs aimed at practicing physicians [3] have begun to fill in additional gaps in the training pipeline for systems medicine.

In the near term systems healthcare must be both pedagogically inter-professional and clinically multi-disciplinary to achieve the full impact of the overall approach. Modifications to existing post-graduate clinical programs are needed and this must be coordinated with the accreditation bodies. Demonstrations of improved clinical and cost effectiveness will be necessary to drive reimbursement reform and ultimately the wide scale adoption. The robustness of systems oriented multimodal data when reduced and made actionable by providers and patients will further stimulate clinical utility and bring about an inflection or tipping point. This future is scheduled to arrive soon at clinic near you.

## Additional file

Additional file 1: Systems Healthcare: A holistic paradigm for tomorrow. (DOCX 214 kb)
