# Supplementary material for: Systems healthcare: a holistic paradigm for tomorrow
Source: BMC Syst Biol. 2017 Dec 19;11:142. doi: 10.1186/s12918-017-0521-2 (PMC5738174; doi:10.1186/s12918-017-0521-2)
Supplement: Additional file 1: — Systems Healthcare: A holistic paradigm for tomorrow. (DOCX 214 kb) [file 12918_2017_521_MOESM1_ESM.docx]

Systems Healthcare: A Holistic Paradigm for Tomorrow Fiandaca, et. al.,

**Additional File 1**

**Case Example: Developing a multimodal approach to Alzheimer’s disease risk**

In this theoretical illustration, we consider a 35 year-old male, retired United States Navy fighter pilot. He works for a major airline as an international pilot. His family history includes parents of European descent, without history of neurological, endocrinological, or cardiovascular disorders, aged 62 and 60 years, respectively. He is married (without children), college educated [1, 2], and maintains a military fitness level, with daily exercise routines, including running, weight training, and swimming. He is a non-smoker but enjoys daily alcoholic beverages. His diet features occasional vegetarian options, supplemented by grains and fish, but he primarily enjoys a high fat diet [3-5], with ample meat dishes and dairy products. He reports having played football and lacrosse since childhood and sustaining two memorable head injuries during his 20s [6, 7], the first at age 21, while playing rugby with friends at the Naval Academy, and the other at age 26, during an simulated ejection drill at TOP GUN. Both were witnessed injuries for which he underwent complete medical assessments for briefly having lost consciousness with the first incident, without displaying any amnesia, and after 5 minutes of unconsciousness and displaying nearly 30 minutes of retrograde amnesia, requiring hospitalized observation, with the second episode [8, 9]. Blood pressure and physical examinations have been normal, and laboratory studies show normal hepatic and renal function, and blood lipid levels. Genetic analyses on peripheral blood leukocytes indicated a single *APOE ε4* polymorphic allele (the other being *ε3*) [10], the presence of the *TREM2* (trigger receptor expressed on myeloid cells 2) gene variant rs75932628 [11], and increased DNA methylation in a group of genes found to be downregulated in early LOAD [12, 13]. As a result of his international flight schedules he reports some difficulties with jet lag [14, 15], especially trouble getting restful sleep [16-21].

**Analysis.** To summarize the holistic factors that may play a role in this individual’s risk of developing LOAD, the clinical and laboratory information can be gathered and initially formulated into a simple risk network (**Figure 3A**). Unfortunately, we are at a point where the specific network nodes and edges remain to be fully defined and their individual significance appropriately weighted. Recent attempts at modeling AD using systems approaches [22, 23] help define the useful quantitative methodologies and begin to display the current limitations based on the assumptions applied, especially regarding the proposed etiology and key factors for consideration. Ultimately, using mathematical systems biology approaches [24-27], a personal model related to LOAD risk may be attainable, and a clinically relevant risk score generated. Such a quantity would be based on the assimilation and interpretation of multifaceted internal datasets (from ‘omics’), including their diverse, time-dependent, multidimensional interactions, while also accounting for the external influences known to modify specific intrinsic physiological expressions (**Figure 3B**), such as the metabolome’s influence on the epigenome and subsequent gene expression.

Potential impactful adjustments to the example case presented could include altering dietary choices (e.g., avoidance of high fat diet), preventing further head injuries, and attempting to improve sleep via job modifications that reduce phase shifting (jet lag). Based on current knowledge, these relatively minor adjustments in behavior may mitigate a degree of the individual’s future risk. Unfortunately, we have not yet acquired the requisite knowledge and experience to successfully understand strategies that may modify the major LOAD risk factors (e.g., APOE, TREM2, DNA methylation) highlighted in the case.

The scenario presented approaches a near state-of-the-art preliminary medical evaluation that incorporates disparate information from a variety of perspectives and available technologies, but still fails to describe a truly holistic approach to the patient. Many additional data elements could be added, including a variety of neuroimaging (e.g., positron emission tomography, functional magnetic resonance imaging) and cerebrospinal fluid measures (e.g., amyloid beta (Aβ), tau species). There are many reasons, both theoretical and practical why this is so. Despite using a carefully constructed case, we remain unclear as to what the holistic clinical determinants might be. Our simplistic perspective proposes that specific genetic risk information related to LOAD (e.g., APOE and TREM2) are static, and that additional knowledge regarding epigenetic modulation of such factors may be inconsequential to future risk determination. This might not be the case. The potential interactions between APOE and TREM2 [28] might contribute to a different interpretation of risk for our example patient. Given such a novel genetic interactions, what about additional influences by other nodes within the same pathway or adjacent pathways? What about potential post-translational or other regulatory influences on their specific gene products? Such considerations and many more may be relevant to the combinatorial nodal influences within the APOE-TREM2 network and many others. We are not too early to consider these complexities, despite currently lacking the capacity to define the particular details. There is a growing appreciation, however, that the proposed multidimensional network relationships and influences (**Figure 3B**) may translate into a variety of modifiable internal and external loci. In regards to APOE and TREM2, those altered influences may be ultimately reflected within a specific cell type (e.g., microglia) considered relevant to the disease pathobiology.

As of today, we remain unsure of the practicality of attempted interpretation of a simplistic network (**Figure 3A**) over a more complex one (**Figure 3B**), despite the general trend towards more holistic approaches. As far as the systems biology of specific diseases (let alone “health”), we currently exist in a knowledge-building mode. We are better able to interpret relationships at specific ‘omic’ levels of interactions and are beginning to appreciate certain preliminary relationships between ‘omic’ layers. We remain far from comprehending the actual interactions that contribute to the “systems biology” of an individual. Despite this seemingly distant goal, there is growing acceptance that such knowledge is required for the ultimate understanding of complex organisms, diseases, and organizations. Importantly, it is essential to begin integrating the disparate sets of information accessible through a single patient, and expanding our understanding of multiomic matrices. Information accumulated through such a personalized systems approach can be combined to provide a more refined representation of cohorts, and eventually populations. In some future time, we anticipate gathering enough personal ‘omic’ information, from enough individuals, fully integrating their internal and environmental influences, and better defining a specific molecular and clinical phenotype. At such a point, we will be positioned to build an integrated personalized approach to the management of health and disease. Until all of these data elements and influences can be adequately combined and analyzed, our modeling capabilities related to systems biology will remain limited, far short of the holistic goal of interpreting the health status of a human being.

Can we propose a more detailed design for such a holistic approach? The first step likely requires an assessment of the biological underpinning for each of the multiomic layers so far identified (**Figure 3B**). Currently, the *DNA/genome structure* remains a major entry point to an understanding of the system. In the example case, since both APOE and TREM2 variants, independently, have been associated with increased LOAD risk, a “risk score” can, in principle, be attributed to each variant. However, while the epidemiological implications of a single APOE ε4 allele are widely known, the definitive mechanistic role of this genetic variant remains elusive. The suggestion of a possible role for TREM2, and nearly two dozen other contributors to LOAD susceptibility and risk, via a combined genetic predisposition provides a much more recent concern. The putative mechanism behind the risk profile for TREM2 remains under intense investigation but points toward a homeostatic role within the microglia. The APOE-TREM2 association appears to regulate a neurodegenerative microglial phenotype switch (MGnD), in combination with other gene influences [29], advancing such relationships towards the *transcriptomic level*. Unfortunately, not only is our current knowledge of this novel association limited, but most current investigations persist in utilizing animal models, rather than investigating humans, with the persistent challenges to human translation that this poses. Likewise, there is a paucity of knowledge regarding the epigenetic regulatory state for this particular set of genes and their downstream pathways. As presented, complexities arise not only when addressing nodal influences existing *within* each ‘omic’ layer, but are exponentially more complex and inherently difficult to define when occurring *between* layers. Although we appear to be approaching an understanding of the complexity associated with how the *Epigenome* influences the *Transcriptome,* and may provide added relevance to the *Genome,* *Proteome*, and *Metabolome,* a comprehensive systems biological integration of these multidimensional layers remains beyond our current capabilities. A glimpse at this future potential will hopefully energize all of us to strive for such goals.

1. Letenneur L, Launer LJ, Andersen K, Dewey ME, Ott A, Copeland JR, Dartigues JF, Kragh-Sorensen P, Baldereschi M, Brayne C *et al*: **Education and the risk for Alzheimer's disease: sex makes a difference. EURODEM pooled analyses. EURODEM Incidence Research Group**. *Am J Epidemiol* 2000, **151**(11):1064-1071.

2. Sharp ES, Gatz M: **Relationship Between Education and Dementia An Updated Systematic Review**. *Alz Dis Assoc Dis* 2011, **25**(4):289-304.

3. Mendoza J, Pevet P, Challet E: **High-fat feeding alters the clock synchronization to light**. *J Physiol* 2008, **586**(24):5901-5910.

4. Coomans CP, van den Berg SA, Houben T, van Klinken JB, van den Berg R, Pronk AC, Havekes LM, Romijn JA, van Dijk KW, Biermasz NR *et al*: **Detrimental effects of constant light exposure and high-fat diet on circadian energy metabolism and insulin sensitivity**. *FASEB J* 2013, **27**(4):1721-1732.

5. Asher G, Sassone-Corsi P: **Time for food: the intimate interplay between nutrition, metabolism, and the circadian clock**. *Cell* 2015, **161**(1):84-92.

6. Plassman BL, Havlik RJ, Steffens DC, Helms MJ, Newman TN, Drosdick D, Phillips C, Gau BA, Welsh-Bohmer KA, Burke JR *et al*: **Documented head injury in early adulthood and risk of Alzheimer's disease and other dementias**. *Neurology* 2000, **55**(8):1158-1166.

7. Shively S, Scher AI, Perl DP, Diaz-Arrastia R: **Dementia resulting from traumatic brain injury: what is the pathology?** *Arch Neurol* 2012, **69**(10):1245-1251.

8. Gottlieb S: **Head injury doubles the risk of Alzheimer's disease**. *Br Med J (Clin Res Ed)* 2000, **321**(7269):1100-1100.

9. Stern RA, Riley DO, Daneshvar DH, Nowinski CJ, Cantu RC, McKee AC: **Long-term consequences of repetitive brain trauma: chronic traumatic encephalopathy**. *PM & R : the journal of injury, function, and rehabilitation* 2011, **3**(10 Suppl 2):S460-467.

10. Liu CC, Kanekiyo T, Xu H, Bu G: **Apolipoprotein E and Alzheimer disease: risk, mechanisms and therapy**. *Nat Rev Neurol* 2013, **9**(2):106-118.

11. Lill CM, Rengmark A, Pihlstrom L, Fogh I, Shatunov A, Sleiman PM, Wang LS, Liu T, Lassen CF, Meissner E *et al*: **The role of TREM2 R47H as a risk factor for Alzheimer's disease, frontotemporal lobar degeneration, amyotrophic lateral sclerosis, and Parkinson's disease**. *Alzheimer's & dementia : the journal of the Alzheimer's Association* 2015, **11**(12):1407-1416.

12. Parachikova A, Agadjanyan MG, Cribbs DH, Blurton-Jones M, Perreau V, Rogers J, Beach TG, Cotman CW: **Inflammatory changes parallel the early stages of Alzheimer disease**. *Neurobiol Aging* 2007, **28**(12):1821-1833.

13. Horvath S, Zhang Y, Langfelder P, Kahn RS, Boks MP, van Eijk K, van den Berg LH, Ophoff RA: **Aging effects on DNA methylation modules in human brain and blood tissue**. *Genome Biol* 2012, **13**(10):R97.

14. Bedrosian TA, Nelson RJ: **Timing of light exposure affects mood and brain circuits**. *Translational psychiatry* 2017, **7**(1):e1017.

15. Lee A, Galvez JC: **Jet lag in athletes**. *Sports Health* 2012, **4**(3):211-216.

16. Dijk DJ, Duffy JF, Czeisler CA: **Circadian and sleep/wake dependent aspects of subjective alertness and cognitive performance**. *J Sleep Res* 1992, **1**(2):112-117.

17. Duffy JF, Kronauer RE, Czeisler CA: **Phase-shifting human circadian rhythms: influence of sleep timing, social contact and light exposure**. *J Physiol* 1996, **495 ( Pt 1)**:289-297.

18. LeVault KR, Tischkau SA, Brewer GJ: **Circadian Disruption Reveals a Correlation of an Oxidative GSH/GSSG Redox Shift with Learning and Impaired Memory in an Alzheimer's Disease Mouse Model**. *J Alzheimers Dis* 2016, **49**(2):301-316.

19. Iliff JJ, Wang M, Liao Y, Plogg BA, Peng W, Gundersen GA, Benveniste H, Vates GE, Deane R, Goldman SA *et al*: **A paravascular pathway facilitates CSF flow through the brain parenchyma and the clearance of interstitial solutes, including amyloid beta**. *Sci Transl Med* 2012, **4**(147):147ra111.

20. Xie L, Kang H, Xu Q, Chen MJ, Liao Y, Thiyagarajan M, O'Donnell J, Christensen DJ, Nicholson C, Iliff JJ *et al*: **Sleep drives metabolite clearance from the adult brain**. *Science* 2013, **342**(6156):373-377.

21. Lucey BP, Bateman RJ: **Amyloid-beta diurnal pattern: possible role of sleep in Alzheimer's disease pathogenesis**. *Neurobiol Aging* 2014, **35 Suppl 2**:S29-34.

22. Hao W, Friedman A: **Mathematical model on Alzheimer's disease**. *BMC systems biology* 2016, **10**(1):108.

23. Calderone A, Formenti M, Aprea F, Papa M, Alberghina L, Colangelo AM, Bertolazzi P: **Comparing Alzheimer's and Parkinson's diseases networks using graph communities structure**. *BMC systems biology* 2016, **10**:25.

24. Nalls MA, McLean CY, Rick J, Eberly S, Hutten SJ, Gwinn K, Sutherland M, Martinez M, Heutink P, Williams NM *et al*: **Diagnosis of Parkinson's disease on the basis of clinical and genetic classification: a population-based modelling study**. *Lancet Neurol* 2015, **14**(10):1002-1009.

25. Chouraki V, Reitz C, Maury F, Bis JC, Bellenguez C, Yu L, Jakobsdottir J, Mukherjee S, Adams HH, Choi SH *et al*: **Evaluation of a Genetic Risk Score to Improve Risk Prediction for Alzheimer's Disease**. *J Alzheimers Dis* 2016, **53**(3):921-932.

26. Harrison TM, Mahmood Z, Lau EP, Karacozoff AM, Burggren AC, Small GW, Bookheimer SY: **An Alzheimer's Disease Genetic Risk Score Predicts Longitudinal Thinning of Hippocampal Complex Subregions in Healthy Older Adults**. *eNeuro* 2016, **3**(3).

27. Escott-Price V, Shoai M, Pither R, Williams J, Hardy J: **Polygenic score prediction captures nearly all common genetic risk for Alzheimer's disease**. *Neurobiol Aging* 2017, **49**:214 e217-214 e211.

28. Krasemann S, Madore C, Cialic R, Baufeld C, Calcagno N, El Fatimy R, Beckers L, O'Loughlin E, Xu Y, Fanek Z *et al*: **The TREM2-APOE Pathway Drives the Transcriptional Phenotype of Dysfunctional Microglia in Neurodegenerative Diseases**. *Immunity* 2017, **47**(3):566-581 e569.

29. Pimenova AA, Marcora E, Goate AM: **A Tale of Two Genes: Microglial Apoe and Trem2**. *Immunity* 2017, **47**(3):398-400.
